# Supplementary material for: Dog Owners’ Survey reveals Medical Alert Dogs can alert to multiple conditions and multiple people
Source: PLoS One. 2021 Apr 14;16(4):e0249191. doi: 10.1371/journal.pone.0249191 (PMC8046193; doi:10.1371/journal.pone.0249191)
Supplement: S1 Table — (DOCX) [file pone.0249191.s001.docx]

**S1 Table. The top six most frequently reported conditions dogs alert to and for dogs that alert to those conditions, the other conditions they are also reported to alert.**

| **Condition** | **Other Conditions to Which Dog Alerts (number of people reported their dog also alerts to this condition)** | |
| --- | --- | --- |
| Anxiety | Migraine (14) | Cancer (1) |
|  | Seizure (6) | Heart complications (1) |
|  | Hypoglycaemia (6) | Pancreatitis (1) |
|  | POTS^*^ (6) | Knee injury (1) |
|  | Narcolepsy (3) | Ankle sprain (1) |
|  | Asthma (3) | Depression (1) |
|  | Periodic paralysis (3) | Syncope (1) |
|  | Hyperglycaemia (2) | PTSD^†^ (1) |
|  | Allergic reaction (2) | Postural hypotension (1) |
|  | Dissociative episodes (2) | Cataplexy (1) |
|  | Arthritis (2) | Ehlers-Danlos Syndrome (1) |
|  | Muscle spasms (2) | Blackouts (1) |
| Hypoglycaemia | Hyperglycaemia (17) | Cancer (1) |
|  | Anxiety (6) | Dystonic storm (1) |
|  | Migraine (4) | Heart complications (1) |
|  | Allergic reaction (3) | Pancreatitis (1) |
|  | Arthritis (2) | Muscle spasms (1) |
|  | POTS^*^ (2) | Sepsis (1) |
|  | Narcolepsy (1) | Low oxygen (1) |
|  | Dissociative episodes (1) |  |
| Hyperglycaemia | Hypoglycaemia (17) | Dystonic storm (1) |
|  | Allergic reactions (2) | Muscle spasms (1) |
|  | Migraine (2) | Sleep apnea (1) |
|  | Anxiety (2) | Sinus tachycardia (1) |
|  | Narcolepsy (1) | POTS^*^ (1) |
|  | Arthritis (1) |  |
| Migraine | Anxiety (14) | Heart complications (1) |
|  | POTS^*^ (5) | Pancreatitis (1) |
|  | Hypoglycaemia (4) | Syncope (1) |
|  | Seizure (3) | PTSD^†^ (1) |
|  | Narcolepsy (2) | Postural hypotension (1) |
|  | Asthma (2) | Cataplexy (1) |
|  | Dissociative episodes (2) | Cluster headaches (1) |
|  | Hyperglycaemia (2) | Ehlers-Danlos Syndrome (1) |
|  | Allergic reaction (2) | Blackouts (1) |
|  | Periodic paralysis (1) |  |
| Seizure | Anxiety (6) | Dissociative episodes (1) |
|  | POTS^*^ (4) | Arthritis (1) |
|  | Hypoglycaemia (3) | Pancreatitis (1) |
|  | Migraine (3) |  |
| POTS | Anxiety (6) | Dissociative episodes (1) |
|  | Migraine (5) | Heart complications (1) |
|  | Seizure (4) | Pancreatitis (1) |
|  | Dystonic storm (2) | Sepsis (1) |
|  | Hypoglycaemia (2) | Low oxygen (1) |
|  | Allergic reaction (1) | Syncope (1) |
|  | Narcolepsy (1) | Cataplexy (1) |
|  | Asthma (1) | Ehlers-Danlos Syndrome (1) |
|  | Addison's Disease (1) | Hyperglycaemia (1) |
|  | Periodic paralysis (1) |  |

*POTS: Postural Orthostatic Tachycardia Syndrome, ^†^ Post Traumatic Stress Disorder
