# Supplementary material for: Dog Owners’ Survey reveals Medical Alert Dogs can alert to multiple conditions and multiple people
Source: PLoS One. 2021 Apr 14;16(4):e0249191. doi: 10.1371/journal.pone.0249191 (PMC8046193; doi:10.1371/journal.pone.0249191)
Supplement: S2 Table — (DOCX) [file pone.0249191.s002.docx]

**S2 Table. The results of Fishers exact tests for sociodemographic variables of the target person and dog and whether or not the dog alerted to multiple conditions, multiple people, or both.**

| **Independent Variable** | **Dependent Variable** | | | | | |  |
| --- | --- | --- | --- | --- | --- | --- | --- |
|  | **Does dog alert to multiple conditions** | | **Does dog alert to multiple people** | | **Does dog alert to both multiple conditions and multiple people** | | |
|  | **Fishers Exact Test Sig. Value (2-sided)** | | | | | |  |
| Is the dog formally trained for medical alert | 0.731 | 0.073 | | 0.126 | |  |  |
| Who trained the dog | 0.762 | 0.083 | | 0.504 | |  |  |
| Does the dog receive maintenance training | 0.732 | 0.451 | | 0.437 | |  |  |
| Who does the maintenance training | 0.586 | 0.214 | | 0.15 | |  |  |
| Gender of target person to whom dog alerts | 0.032 | 0.488 | | 0.488 | |  |  |
| Age of target person to whom dog alerts | 0.09 | 0.25 | | 0.139 | |  |  |
| Sex of dog | 0.301 | 0.611 | | 1 | |  |  |
| Dog purebred or mixed breed | 0.429 | 0.375 | | 1 | |  |  |
| How long target person has been with their dog | 0.228 | 0.121 | | 0.199 | |  |  |
| Target person's feelings towards dog | 0.096 | 0.519 | | 0.759 | |  |  |
| Friendliness of dog towards people other than target person | 0.771 | 0.937 | | 0.753 | |  |  |
| Time with dog before it began alerting | **0.004** | 0.84 | | 0.391 | |  |  |
| Is the dog trained for other specialised activities | 0.29 | 0.119 | | 0.283 | |  |  |

***With Šidák correction, α_SID_ = .004
