# Supplementary material for: Dog Owners’ Survey reveals Medical Alert Dogs can alert to multiple conditions and multiple people
Source: PLoS One. 2021 Apr 14;16(4):e0249191. doi: 10.1371/journal.pone.0249191 (PMC8046193; doi:10.1371/journal.pone.0249191)
Supplement: S3 Table — (DOCX) [file pone.0249191.s003.docx]

**S3 Table. The conditions that dogs alerted other people that were different from the conditions to which the dog alerted the target person.**

| **Dog** | **Conditions to which dog alerts primary person** | **Conditions to which dog has alerted other people** |
| --- | --- | --- |
| 1 | Periodic paralysis | Knee injury |
|  | Anxiety | Ankle sprain |
|  |  | Shoulder spasm |
| 2 | Allergic reaction | Heart attack |
|  | Narcolepsy | High blood pressure |
|  | Asthma |  |
|  | Paralytic episodes |  |
|  | Anxiety |  |
|  | Migraine |  |
|  | POTS^*^ |  |
|  | Cataplexy |  |

*POTS: Postural Orthostatic Tachycardia Syndrome
