# Supplementary material for: Dog Owners’ Survey reveals Medical Alert Dogs can alert to multiple conditions and multiple people
Source: PLoS One. 2021 Apr 14;16(4):e0249191. doi: 10.1371/journal.pone.0249191 (PMC8046193; doi:10.1371/journal.pone.0249191)
Supplement: S1 File — (DOCX) [file pone.0249191.s004.docx]

PLOS ONE

Manuscript submission

Re: Personal Communications

To Whom it May Concern,

The manuscript submitted contains personal communication cited on line 104. This communication was with Olivia Rockaway in February of 2020. Olivia is a trainer of diabetic alert dogs and, at the time, was a master’s student at Queen’s University Belfast conducting research in the lab supervised by Dr Catherine Reeve. The personal communication was in relation to the phenomenon being documented in the submitted manuscript, specifically, that Olivia had recently documented an instance where a dog that that had been successfully trained to alert to her own hypoglycaemia spontaneously alerted to a friend of hers that was experiencing severe anxiety, and at another time, an asthma attack.

It should be noted that C. Reeve has had previous conversations with owners of medical alert dogs who report the same phenomenon of the dog alerting to multiple conditions and multiple people. The conversation with Olivia Rockaway was the most recent and most carefully documented conversation.

Please do not hesitate to contact me if you need further support for the communications described.


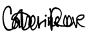


Catherine Reeve (corresponding author)

Lecturer, Animal Welfare and Behaviour

School of Psychology

Queen’s University Belfast
