# Supplementary material for: Dog Owners’ Survey reveals Medical Alert Dogs can alert to multiple conditions and multiple people
Source: PLoS One. 2021 Apr 14;16(4):e0249191. doi: 10.1371/journal.pone.0249191 (PMC8046193; doi:10.1371/journal.pone.0249191)
Supplement: Attachment — Submitted filename: Reeve at al. - Response to Reviewers.docx [file pone.0249191.s007.docx]

I Anna S Olsson, Ph.D.
Academic Editor
PLOS ONE

Re: PONE-D-20-32952 Response to Reviewers

Dear Dr Olsson,

Thank you and the reviewer for taking the time to review our manuscript. Thank you for taking the time to review our manuscript and have a reviewer provide feedback. We are pleased to have an opportunity to revise our manuscript once more. In the revised manuscript, we have carefully considered both you and the reviewer’s comments and suggestions and have addressed and/or responded to each of the points raised. The feedback was very helpful overall, and we are appreciative of such constructive feedback on our original submission. After addressing the issues raised, we feel the quality of the paper is much improved.

Please note that in our previous submission we neglected to address the comments/edit suggestion made by yourself, the editor. Therefore, we have addressed the previous comments as well as the most recent comments together here.

**Response to Editor Comments**

Comment:

Please ensure that your manuscript meets PLOS ONE's style requirements, including those for file naming.

Response:

We have made formatting and style edits to the title page and manuscript according to the PLOs ONE style template.

Comment:

In your Methods section, please provide additional information about the participant recruitment method and the demographic details of your participants. Please ensure you have provided sufficient details to replicate the analyses such as: a) the recruitment date range (month and year), b) a description of any inclusion/exclusion criteria that were applied to participant recruitment, c) a table of relevant demographic details, d) a description of how participants were recruited, and e) descriptions of where participants were recruited and where the research took place.

Response:

A) Recruitment date range was added on Line 126.
B) Inclusion criteria were explicitly identified through the addition of a header on Line 128
C) Demographic details can be found in Table 1.
D) Recruitment details were explicitly identified through the addition of a header on Line 123.
E) Where participants were recruited from was flagged by the reviewer in the previous round of revisions. This information has been added in Line 124. We also clarified that the survey was completed by participants on their own electronic device on Line 186.

Comment:

Please review your reference list to ensure that it is complete and correct. If you have cited papers that have been retracted, please include the rationale for doing so in the manuscript text, or remove these references and replace them with relevant current references. Any changes to the reference list should be mentioned in the rebuttal letter that accompanies your revised manuscript. If you need to cite a retracted article, indicate the article’s retracted status in the References list and also include a citation and full reference for the retraction notice.

Response:

We have carefully reviewed all of our references and can confirm that they are accurate and that none have since been retracted. This is further confirmed by the fact that all papers have active DOIs (which have been added to the references as is highlighted in the revised manuscript)

**Response to Reviewer Comments**

Comment:

Among the limitations of the study, please add a reflection on that the data are owner-reported, thus strictly speaking it is about *owners perceiving* their dogs to be alerting to multiple conditions. Given what you write on lines 510-511, "Given the results of other studies which found discrepancies between owner reports and objective assessments of MADs", it is important to mention this as a study limitation.

Response:

Thank you for identifying this important point. We have added Lines 570-573 which highlight this aspect of the study and caution the reader to interpret the results accordingly.

Comment:

Since this survey was only done in English and distributed worldwide via the snowballing technique, how sure are you that the respondents indeed mastered the English language enough to understand your questions? Also, could cultural differences have an impact on the responses to your specific questions? It might be good to add a few lines about this in your discussion.

Response:

Regarding the first point, we did not require that participants indicate that they had mastered the English language at any point in the survey. We did require, however, that participants read the participant information sheet and then consent to a series of statement prior to beginning the survey. We presume that anyone who could not comprehend the information sheet and the consent statements would not have consented to participating.

We do not have any reason to believe that participants’ responses to the questions themselves could be affected by cultural differences. We did, however, add a comment about the potential for participants’ interpretation of their dogs’ behaviour to be different across cultures, as supported by the addition of another reference (64, Amici et al., 2019).

We hope these changes are suitable. We feel that the revised manuscript is much stronger after making your recommended changes. Thank you for your time,

Dr Catherine Reeve
Animal Welfare and Behaviour
School of Psychology
Queen’s University Belfast
