# Supplementary material for: Dog Owners’ Survey reveals Medical Alert Dogs can alert to multiple conditions and multiple people
Source: PLoS One. 2021 Apr 14;16(4):e0249191. doi: 10.1371/journal.pone.0249191 (PMC8046193; doi:10.1371/journal.pone.0249191)
Supplement: Attachment — Submitted filename: Reeve et al. - Response to Reviewer Comments.docx [file pone.0249191.s006.docx]

I Anna S Olsson, Ph.D.
Academic Editor
PLOS ONE

Re: PONE-D-20-32952 Response to Reviewers

Dear Dr Olsson,

Thank you for taking the time to review our manuscript and have a reviewer provide feedback. We are pleased to have an opportunity to revise our manuscript. In the revised manuscript, we have carefully considered the reviewers comments and suggestions and have addressed and/or responded to each of their points. The reviewer’s comments were very helpful overall, and we are appreciative of such constructive feedback on our original submission. After addressing the issues raised, we feel the quality of the paper is much improved. Following the reviewer’s suggestions, we have added three additional tables as supplementary materials (file entitled: S1 S2 S3 Tables), the captions for which can be found at the very end of the manuscript file.

Upon closer inspection of the manuscript, we have also made a few further edits (i.e., editing references that were formatted incorrectly). All changes are indicated with track changes in the file named Reeve et al – Revised Manuscript with Track Changes.doc.

Thank you for your time and we look forward to hearing from you.

Sincerely,

Dr Catherine Reeve

**Response to Reviewer Comments**

Dear Reviewer,

Thank you for taking the time to review our manuscript, *Medical alert dogs are alerting to multiple conditions and multiple people*. We very much appreciate your feedback. You highlighted a number of ways in which we could improve the quality of our manuscript, and we have made the changes accordingly. Please find our responses to your comments below:

**Major Comments**

Comment:

The authors document sociodemographic information for MAD, but why did they choose not to examine whether people other than the primary person, if dogs alerted to multiple people/multiple conditions, actually had a condition and which one that was?

Response:

Thank you for your query. We would like to point out that in lines 169-172 (in the revised manuscript **without track changes**), participants were asked, to the best of their knowledge, what conditions their dog had alerted to other people. We did not ask participants to report specifically what conditions the dogs were alerting to specific people and therefore cannot report this level of detail. But we agree that the findings related to the conditions that dogs alert other people to were not discussed in much detail and, therefore, the conditions to which dogs were reported to alert to other people have now been presented in Table 7.

Furthermore, in lines 291-295 we state that 94% of participants reported that if the dog was alerting to other people, it was alerting the same condition(s) to which the dog alerted the target person. Only 6% (n=2) of those who reported that their dog alerted to other people reported that their dog alerted other people to a different condition. In response to your comment, we have added the details of those who reported that their dog alerted other people to conditions other than those conditions to which the dog alerted the primary person in a table in the supplementary materials (S3) and have discussed these findings further in the discussion (Lines 483-497).

Comment:

When dogs also alerted to other conditions, it would be interesting to examine which these conditions were. Given that there many conditions that were reported infrequently, I would focus this analysis on the more common ones (anxiety, hypoglycaemia, hyperglycaemia, seizure and POTS). This could be a descriptive analysis, not an inferential one, but it would allow to remove some speculation that currently occurs in the discussion.

Response:

You have raised an important point and we agree that knowing which other conditions dogs alert to would be a valuable addition to the manuscript. A supplementary table (S1 Table) has been added that presents the top six most frequently reported conditions that dogs alert to and, for dogs that alert to those conditions, the other conditions they are reported to alert. We have also conducted further analyses to examine whether there are any significant associations between whether or not a dog alerts to each of these conditions and whether or not the dog alerts to multiple conditions (see response to next comment).

Comment:

The paper does not report any test statistics, degrees of freedom, or P-values. I would like to see these added to substantiate the claims made by the authors.

Response:

Thank you for raising an important point. The only inferential statistics performed were Fisher’s exact tests because comparisons were either 2x2 tables, or, for tables larger than 2x2, one or more of the cells in the table contained counts less than 5 (based on the assumptions of Chi-Square tests of independence as discussed by McHugh, 2013). When using Fisher’s exact tests only the p value is reported.

The authors had previously discussed included binomial logistic regression to analyse whether any of the variables in Table 1 predicted whether or not a dog alerted to multiple conditions, multiple people, or both multiple conditions and multiple people, but considering that a basic assumption of regression analyses is that there are roughly 12 participants per independent variables, our small sample size led us to decide that the results of such analyses would be not be valid.

But considering the comments about presenting more detailed analyses of the top six conditions to which dogs were reported to alert, additional analyses were included. We analysed whether alerting to one of the top six conditions was associated with a greater likelihood of alerting to other conditions, other people, or both other conditions and other people. Results revealed that there were no significant relationships between the variables. These results are now reported on lines 238-242, 285-289, and 311-314.

Comment:

In the statistical analysis, many comparisons have been made. Because of this, significant findings can occur by chance. A more conservative way is to correct for the number of statistical tests done by dividing the cut-off value (P-value) by the number of tests run. The authors did not choose to do this, but it should be revised by a statistician whether it is not more correct to do so still.

Response:

Thank you for highlighting this error on our part. We have adjusted our analyses and used a Šidák correction for multiple comparisons. As a result of the correction for multiple comparisons, one of our previously significant findings is no longer significant (association between gender of target person and whether or not the dog alerts to multiple conditions) and as such, discussion of this finding has been removed.

**Minor Comments**

**Introduction**

- Line 46: reference 1 is incomplete in the list of references. Please correct.

>> Reference 1 (now on line 558) has been completed.

- Line 54: in addition to reference 14, there is also the work by Martos Martinez-Caja (2019 in Epilepsy & Behavior), which is more recent, to support this.

>> Thank you very much for pointing out this relevant paper. We have included the paper as a citation on line 54 and it is included in the reference list (number 15, line 595).

- Line 88: I do not know the word ‘brinsel’. I have looked it up, but could not retrieve its meaning. Can this word be substituted, if not explained?

>> We appreciate that this term is not one that is commonly used outside of medical detection dog training. We have also identified that it was, in fact, spelled incorrectly. We have therefore edited the spelling of the word accordingly, and have further included a brief definition on lines 89-90.

- Line 93: “It is possible that certain aspects of their training”. Replace “their” by “the dogs’ “, so that it is more clear.

>> The recommended edit has been made on line 94.

- Line 107: typo – “on” should be “an”

>> This error has been corrected on line 108.


**Materials and Methods**

- Lines 120-125 and line 128: in which language was the survey designed? For a UK target audience? Is anything known about the country of origin of the participants?

>> Thank you for pointing out the lack of detail regarding the survey itself and the participants. The survey was only available in English and available to participants worldwide. We have added this information as well as general descriptive analyses of the locations of participants on lines 122, 126. 203-205.

- Line 129-130: rather than having people ask for the survey, I would suggest to include it in full as supplementary material

>> You have raised an important point however, we feel that it would be best to maintain that the survey be available only upon request, simply because the survey flow is quite complex. The survey was designed to have four major branches depending upon the nature of the participants’ relationship with the medical alert dog and it is therefore a long and complicated-looking document. If the editor and reviewer do not consider the length and flow of the survey to be an issue, we will be happy to provide the survey itself.

- Line 167: To make this sentence easier to read, I would add “for” between “knowledge,” and “what condition(s)”

>> This edit has been made on line 170.

- Line 187: typo – “altered” should be “alerted”

>> This error has been corrected on line 190.

**Results**

- Table 2: this is a very important table, that could be even more informative if it was also added how many dogs were trained/untrained when they alert to a particular condition.

While we agree that this would be a very interesting addition to the table, unfortunately it is not possible to add this information. When participants with trained MADs were asked which condition their dog was first trained to alert to, they often also included the other conditions to which the dog began alerting in their response. Therefore, we are unable to differentiate between conditions for which the dog was trained and those that it was not trained for but began alerting.

- Also, a table or figure should be able to stand on its own, meaning everything in it must be clear. Therefore, I would suggest explaining the abbreviations POTS and PTSD below the table.

>> Thank you for pointing out this error on our part. Table 2 has been edited accordingly and tables added since review have also included these footnotes (including the supplementary materials).

- Line 248: the numbers here (90% and 10%) do not correspond to those in table 5. Please check and adjust as necessary.

>> The numbers you are referring to are now found in Table 3 and Table 4. In Table 3, we would like to bring your attention to the variable “Dog alerts to multiple conditions”. In this table, the total number of participants that reported that their dog does **not** alert to multiple conditions is 10. In Table 5, the total number of dogs that are reported to alert to **single condition** is also 10. Therefore, we are unable to identify any errors in the data between these tables. If we have incorrectly identified your specific area of concern, please do not hesitate to respond accordingly.

- Lines 279-281: I would break this down for the most commonly alerted conditions as well.

>> We agree that this would be a valuable addition to the manuscript. We have therefore added Table 7, which presents the conditions to which dogs were reported to alert other people from most commonly reported to least commonly reported. We also conducted further analyses to examine whether or not dogs that alerted to these top six conditions were associated with whether or not a dog alerted to multiple people (Lines 285-289). Lastly, we also included further information in the supplementary material (S3 Table), which presents the data for the two dogs that were reported to alert other people to conditions other than those conditions they dog alerted to the target person.

**Discussion**

- Line 316 and further: this discussion could be substantiated if the authors examined their data in a bit more detail for the most commonly reported conditions that dogs alert to (see major comment). At the moment it seems that dogs mainly alert the same condition in other people in the household, but surely that is more common for some conditions than others, since it is unlikely e.g. that two people from the same household have epilepsy. Although I do appreciate the conciseness with which the authors present their results, a more detailed presentation of the data would help here.

We appreciate the reviewer highlighting an important area where we could substantiate our claims. Additional analyses revealed no significant associations between whether dogs alerted anxiety and whether or not they alerted to other conditions, people, or both. But considering the conditions to which dogs were reported to alert to other people, further descriptive analyses revealed that anxiety was the second most common condition that dog alerted other people. We have further examined the most common conditions to which dogs alerted other people and have presented this data in Table 7. As indicated in the table, anxiety was the second most commonly reported condition that dogs alerted to other people. This point was discussed in the discussion at more length.

- Line 360: “for whether they decide to alert” – I would be careful with this kind of working. A dog does not decide to intentionally alert a person. The dog may decide to intentionally perform a particular behavior (which the owner then interprets as an alert).

>> Thank for you highlighting this point. We agree with your statement that dogs do not have a threshold for whether to decide to alert a person, but rather, have a threshold for whether they perform an alert behaviour. We have changed the wording of this sentence accordingly (line 384).

- Lines 476-477: also, how was the study presented to the respondents? Is there a risk of response bias in that respondents may have felt they were expected to have a dog that responds to multiple conditions/people. Perhaps the authors could elaborate on this as well?

>> Thank you for raising an important point. The information sheet presented to participants before completing the survey did in fact state that researchers were interested in whether or not their dog alerted to multiple conditions or multiple people. This could have resulted in demand characteristics influencing participants’ responses. This point has been discussed on lines 508-515.

We hope these changes are suitable. We feel that the revised manuscript is much stronger after making your recommended changes. Thank you for your time,

Dr Catherine Reeve
Animal Welfare and Behaviour
School of Psychology
Queen’s University Belfast
